# Protective effect of bioactive iridium nanozymes on high altitude-related hypoxia-induced kidney injury in mice

**DOI:** 10.3389/fphar.2023.1115224

**Published:** 2023-02-20

**Authors:** Yujing Wang, Meijun Shi, Zongtang Chu, Xinlin Yan, Guoxing You, Gan Chen, Hong Zhou

**Affiliations:** ^1^ Institute of Health Service and Transfusion Medicine, Academy of Military Medical Sciences, Beijing, China; ^2^ Key Laboratory of Pollution Ecology and Environmental Engineering, Institute of Applied Ecology, Chinese Academy of Sciences, Shenyang, China; ^3^ National Engineering Research Center for the Emergency Drug, State Key Laboratory of Toxicology and Medical Countermeasures, Beijing Institute of Pharmacology and Toxicology, Beijing, China

**Keywords:** acute high-altitude diseases, inflammation, kidney damage, microbiome, metabolites, nanozyme

## Abstract

**Introduction:** High altitude-related hypoxia-induced organ damage significantly impacts people who are exposed to acute high-altitude environment. At present, kidney injury still lacks effective treatment strategies. Iridium nanozymes (Ir-NPs) are a nanomaterial with various enzymatic activities and are expected to be used in kidney injury treatment.

**Methods:** In this study, we simulated a high-altitude environment (6000 m) to induce a kidney injury model, and explored the therapeutic effect of Ir-NPs in mice with kidney injury in this environment. Changes in the microbial community and metabolites were analyzed to explore the possible mechanism underlying the improvement of kidney injury during acute altitude hypoxia in mice treated with Ir-NPs.

**Results:** It was discovered that plasma lactate dehydrogenase and urea nitrogen levels were considerably increased in mice exposed to acute altitude hypoxia compared to mice in a normal oxygen environment. Furthermore, there was a substantial increase in IL-6 expression levels in hypoxic mice; contrastingly, Ir-NPs decreased IL-6 expression levels, reduced the levels of succinic acid and indoxyl sulfate in the plasma and kidney pathological changes caused by acute altitude hypoxia. Microbiome analysis showed that bacteria, such as Lachnospiraceae_UCG_006 predominated in mice treated with Ir-NPs.

**Conclusion:** Correlation analysis of the physiological, biochemical, metabolic, and microbiome-related parameters showed that Ir-NPs could reduce the inflammatory response and protect kidney function under acute altitude hypoxia, which may be related to intestinal flora distribution regulation and plasma metabolism in mice. Therefore, this study provides a novel therapeutic strategy for hypoxia-related kidney injury, which could be applied to other hypoxia-related diseases.

## 1 Introduction

At higher altitudes, the atmospheric environment decreases in pressure and becomes increasingly anoxic. Soldiers, mountain rescue workers, and climbers have an increased risk of altitude disease, which can cause damage to vital organs and even become life-threatening without prompt treatment (Bärtsch and Swenson, 2013). The most commonly reported organ injuries due to acute altitude hypoxia include heart disease, high-altitude cerebral edema, and pulmonary hypertension. Moreover, kidneys are also very susceptible to hypoxia (Zhang et al., 2021c), as the effective blood flow and oxygen supply to kidneys are reduced in the hypoxic environment at high altitude, causing acute kidney injury (Lee et al., 2019; Wang et al., 2022c) and leading to the development and progression of kidney fibrosis, and chronic kidney disease (Shu et al., 2019). Therefore, neglecting adequate renal protection can lead to kidney failure. However, there is still a lack of effective treatment strategies to improve kidney injury. Currently, drugs used to treat acute high-altitude diseases mainly focus on relieving hypoxia and managing the adverse reactions of high altitudes; these include oxygen inhalation and preventive drugs, which cannot fundamentally alleviate the damage. Therefore, there is a growing need for better therapies to mitigate hypoxia-induced injuries.

Previous studies have indicated that acute altitude hypoxia-induced organ damage is primarily due to oxidative stress caused by the increased production of reactive oxygen species (Maiti et al., 2010; Sinha et al., 2010). In addition, the imbalance of intestinal flora is also one of the possible causes of kidney injury. Some studies have found that the intestinal flora remains relatively stable under normal conditions. However, its composition can be affected by extreme environmental conditions such as hypoxia, heat, and cold (Sket et al., 2018; Ramos-Romero et al., 2020). Thus, acute exposure to high-altitude hypoxia can alter the diversity and composition of the gut microbiota (Zhang et al., 2018; Sun et al., 2020). It has been shown that the gut microbiota is closely associated with the occurrence and progression of kidney diseases (Chi et al., 2021; Mosterd et al., 2021; Xu et al., 2021). Intestinal microbiota disruptions and increased intestinal permeability have been shown to lead to bacterial translocation and changes in the associated metabolites (Xu et al., 2022). Therefore, identification of effective antioxidants and anti-inflammatory drugs that regulate intestinal microflora could be a promising strategy for treating high altitude-related kidney injury.

Nanozymes comprise a recently-developed type of nanomaterial that has enzymatic catalytic activity. In 2007, it was discovered that specific metallic or inorganic nanoparticles could directly mimic the catalytic functions of natural enzymes (Wang et al., 2020). As synthetic compounds, nanozymes exhibit long-term antioxidant properties. Compared with natural enzymes, they have the advantages of good stability, low cost, easy preparation, and adjustable activity (Huang et al., 2019; Wu et al., 2019; Zhang and Liu, 2020). Therefore, they have been widely used in biological detection and disease treatment in recent years. Iridium nanoparticles (Ir-NPs) possess both SOD- and CAT-like activities (Liang et al., 2022), and are effective reactive oxygen/nitrogen species scavengers in treating metastatic breast tumors, wound healing, and acute kidney injury (Zhang et al., 2021a; Wang et al., 2022b; Wu et al., 2022). However, the role of Ir-NPs in acute altitude kidney injury treatment has not yet been reported. Therefore, in this study, we investigate the role of synthetic antioxidant Ir-NPs in acute kidney injury and its effect on the host flora and metabolism in a high-altitude environment. We also explored the possible mechanism underlying the improvement of kidney injury under acute altitude hypoxia induced by Ir-NPs using microbiological and metabolomics techniques. Therefore, this work expands the application field of iridium nanozyme and provides a new treatment strategy for kidney injury caused by hypoxia at higher altitudes.

## 2 Materials and methods

### 2.1 Synthesis and characterization of Ir-NPs

Ir-NPs were prepared according to a previously described procedure (Zhang et al., 2021a). In a typical synthesis, iridium trichloride hydrate (26.6 mg) was dissolved in 80 mL of H_2_O. This solution was then mixed with 80 mL of an ethanol solution containing polyvinyl pyrrolidone (PVP) (372 mg) and stirred magnetically. The solution was then incubated overnight at 25°C. After the clear yellow solution was refluxed at 100°C for 6 h, and the solvent was removed using rotary evaporation. Finally, the nanoparticles were characterized using various techniques, as follows. Transmission electron microscopy (TEM, Hitachi H-7800, Japan) images were recorded at 200 kV. In addition, the hybrid bonding state of elemental Ir was analyzed using X-ray photoelectron spectroscopy (XPS, Thermo Fisher Scientific K-Alpha, United States), and data from Fourier transform infrared spectroscopy (FT-IR) were obtained using an ALPHA FTIR Spectrometer (Bruker, Is5, United States).

SOD- and CAT-like activities of Ir-NPs were assessed using the SOD (WST-1 method) and CAT assay kits (Nanjing Jiancheng Bioengineering Institute, Nanjing, China) respectively. WST-1 reacts with superoxide anions (O_2_^−^) catalyzed by xanthine oxidase to produce a water-soluble formazan dye, which possess a characteristic absorption maximum at 450 nm. Since SOD can catalyze superoxide anion disproportionation, this reaction step can be inhibited by SOD. Briefly, 20 µL of Ir-NPs (1 mg/mL) was mixed with 20 µL of enzyme working solution; afterwards, 200 µL of substrate application solution was added. After incubation at 37°C for 20 min, the absorbance was measured at 450 nm using a plate reader. The CAT-like activity of the Ir-NPs (1 mg/mL) was confirmed using the commercial kit according to the manufacturer’s instructions (Nanjing Jiancheng Bioengineering Institute, China) (Zhang et al., 2023).

### 2.2 Animals

Animal experiments were approved by the Institutional Animal Care and Use Committee of the Academy of Military Medical Sciences. This study conformed to the Guide for the Care and Use of Laboratory Animals. Male BALB/c mice (6–8 weeks old) were purchased from Charles River (Beijing, China) and used after 4–6 days of acclimation. Mice were randomized into three groups. The CK (control check) group (*n* = 6) was kept in a normal oxygen environment. The Ns (normal saline) group (*n* = 6) and Ir-NPs group (*n* = 6) were kept at 6,000 m altitude simulation cabin (Guizhou Fenglei). The mice in the Ir-NPs group were gavaged with Ir-NPs (50 mg/kg) in normal saline every day, while those in the NS group were gavaged with the same volume of normal saline. After 7 days, blood samples were collected and assayed immediately. Plasma was obtained *via* centrifugation at 800 ×g for 10 min at 4°C and then frozen at −80°C until further analysis. Afterwards, the mice were sacrificed and the kidneys were removed and washed with normal cold saline, and the cecal content was collected into sterile tubes and stored at −80°C until further analysis.

### 2.3 Analysis of physiological and biochemical parameters

A blood cell analyzer (Mindray, Shenzhen, China) was used to analyze approximately 100 µL of mouse whole blood. In addition, plasma biochemical analysis was performed using a biochemical analyzer (MNCHIP, Tianjin, China).

### 2.4 Histological assays and immunohistochemistry

Mouse kidneys were rinsed with a pre-cooled saline solution and fixed in 4% paraformaldehyde for 24 h before histopathological analysis. Tissue samples were embedded in standard paraffin and cut into 4 μm-thick slices. Subsequently, the slices were dewaxed with xylene and stained with hematoxylin and eosin (H&E). The stained sections were then scanned using a Nano Zoomer Digital Pathology Slicer microscope (Nano Zoomer-XR, C12000, Hamamatsu, Japan). The images were captured using a K-Viewer (KFBIO, China) and analyzed using Image-Pro Plus 6.0 software (Media Cybernetics, United States).

Immunohistochemical analysis of IL-6 expression in kidney tissue sections was also performed. Paraffin sections of mouse kidney tissue were cleaned in normal saline, placed in EDTA (pH 9.0) for antigenic retrieval, and incubated in 3% hydrogen peroxide to block endogenous peroxidase activity. The sections were blocked with 3% BSA (Biosharp, China) and incubated at 25°C for 30 min. Anti-IL-6 antibodies (Servicebio, China) were then added to the sections and incubated at 4°C overnight. Afterward, the sections were washed three times in phosphate buffered saline (PBS) (pH 7.4) (Servicebio) and incubated with the antibodies of HRP (Horse Radish Peroxidase)-labeled goat anti-rabbit IgG for 50 min at 25°C. After washing the sections with PBS, a freshly prepared DAB color development solution was added to the sections. Afterwards, the sections were stained with hematoxylin (Servicebio) for approximately 3 min. The dye was rinsed with running water and the sections were mounted after dehydration. Hematoxylin-stained nuclei appear in blue color, while brownish-yellow DAB-stained sections indicate positive IL-6 expression. The intensity of IL-6 expression was analyzed using Image-Pro Plus 6.0 software (Media Cybernetics, United States).

### 2.5 Bacterial DNA extraction and 16S rRNA amplification

DNA was extracted from 500 mg cecal samples using a PowerSoil DNA Isolation Kit (MoBio Laboratories, Inc., CA, United States). The extracted DNA was tested for quality and concentration using a Nanodrop 2000 instrument (Thermo Fisher Scientific, Inc., United States). The PCR amplification primers 338F (5′-ACT​CCT​ACG​GGA​GGC​AGC​AG-3′) and 806R (5′-GGACTACHVGGGTWTCTAAT-3′) were used to amplify the V3-V4 region of the bacterial 16S rRNA genes. An 8-bp-long barcode sequence was added to the 5′-end of each upstream and downstream primer to differentiate the samples. Each PCR reaction (total volume: 25 μL) contained 12.5 μL 2× Taq Plus Master Mix Ⅱ (Vazyme Biotech Co. Ltd., China), 3 μL BSA (2 ng/μL), 1 μL forward primer (5 μm), 1 μL reverse primer (5 μm), 2 μL DNA (30 ng total DNA added), and 5.5 μL ddH_2_O. The thermocycling conditions were: pre-denaturation at 95°C for 5 min; 28 cycles of denaturation at 95°C for 45 , annealing at 55°C for 50 s, and extension at 72°C for 45 s; and a final extension step at 72°C for 10 min. Amplification was performed on an ABI 9700 PCR instrument (Thermo Fisher Scientific, Inc.), and the PCR products were separated using 1% agarose gels to determine the size of the target band. The PCR products were purified using an Agencourt AMPure XP (Beckman Coulter, Inc., United States) nucleic acid purification kit.

### 2.6 Sequencing and data analysis

The PCR products were used to construct sequencing libraries to assess microbial diversity. An NEB Next Ultra II DNA Library Prep Kit (New England Biolabs, Inc., United States) was used for library preparation. Paired-end sequencing was performed on an Illumina Miseq PE300 high-throughput sequencing platform (Illumina, Inc., United States) at Beijing Aikejia Biotechnology. Fastq data quality control (QC) was performed using trimmomatic. The sliding window strategy was used for trimmomatic sequences with the window size set to 50 bp, average mass value of 20, and minimum retained sequence length of 120. When Pear (v0.9.6) was used for stitching, the minimum overlap was set to 10 bp, and the mismatch rate was set to 0.1. After Mosaic, sequences less than 230 bp in length were removed using Vsearch (v2.7.1) software, and chimeric sequences were removed using the uchime method according to the Gold Database. Operational taxonomic units (OTU) were clustered for high-quality sequences using Vsearch (v2.7.1) software program algorithm, and valid tags with sequence similarities ≥97% were assigned to the same OTUs. The sequence tag with the highest abundance within each OTU cluster was selected as a representative sequence. Subsequently, the BLAST algorithm was used to annotate species classification using the Silva database (Release 138, http://www.arb-silva.de). QIIME (v2.0.0) software was used to analyze the α diversity index (including the Chao1, Simpson, Shannon, PD_whole_tree, Observed_species, and Goods_coverage indices, among others) using the R stats package. The Wilcoxon rank test in ggpubr (v0.4.0) was used to compare the α diversity indices between groups, and *p* < 0.05 was considered significant (Zhang et al., 2021b). QIIME (v2.0.0) software was also used to calculate the beta diversity distance matrix based on species annotation and relative abundance. The weighted Unifrac distance was used to generate a clustering heat map and perform PCoA analysis using R (v3.6.0) software. The R packages ggplot2 (v3.3.2) and vegan were used for data calculation, NMDS analysis, and mapping. Mothur (v1.34.4) software was used to analyze the differences between the metastasis groups, and Python (v2.7) software was used for the LEfSe analysis. Co-occurrence network analysis was performed using the R package psych and visualized using Cytoscape software (v3.7.1). Picrust2 was used for microbial function prediction, and STAMP software was used to analyze differences.

### 2.7 Metabolite extraction and UHPLC-MS/MS analysis

Metabolite extraction and metabolomics analyses were performed using mouse plasma samples. Briefly, 50 mg plasma samples were collected in 1.5 mL Eppendorf microcentrifuge (EP) tubes, and the solution was extracted using 1 mL of 70% methanol as an internal standard. The mixture was shaken for 5 min and left to stand on ice for 15 min. The samples were then centrifuged at 12,000 ×g for 10 min at 4°C. After centrifugation, 400 µL of the supernatant was transferred to the appropriate EP tubes. The samples were stored in a −20°C refrigerator overnight before being centrifuged again at 12,000 ×g for 3 min at 4°C. Following centrifugation, 200 μL of the supernatant was collected and analyzed using UHPLC-MS/MS. UPLC-MS analysis was performed using ultra-performance liquid chromatography (UPLC) (Exion LC AD, https://sciex.com.cn/) and tandem mass spectrometry (MS/MS) (QTRAP^®^, https://sciex.com/). Aliquots of supernatants from all samples were pooled and used as QC samples. Every six test samples during the instrumental analysis were injected with a QC sample to monitor the repeatability and stability of the instrument.

### 2.8 Statistical analysis

The Kruskal—Wallis test was used to obtain the overall *p*-value, while Dunn’s pairwise comparison test was used to obtain the statistical significance of the differences obtained between groups (*, *p* < 0.05; **, *p* < 0.01). The correlations between the main bacterial communities and metabolites were analyzed using canonical correlation analysis. Heat maps of the different metabolites were generated using a heatmap in R. One-way analysis of variance (ANOVA) was used to compare the average optical density among groups, and the error lines indicate the SDs. Results were deemed significantly different at *p* < 0.001 (*, *p* < 0.05; **, *p* < 0.01; ***, *p* < 0.001).

## 3 Results

### 3.1 Synthesis of ultra-small Ir-NPs

**FIGURE 1:**
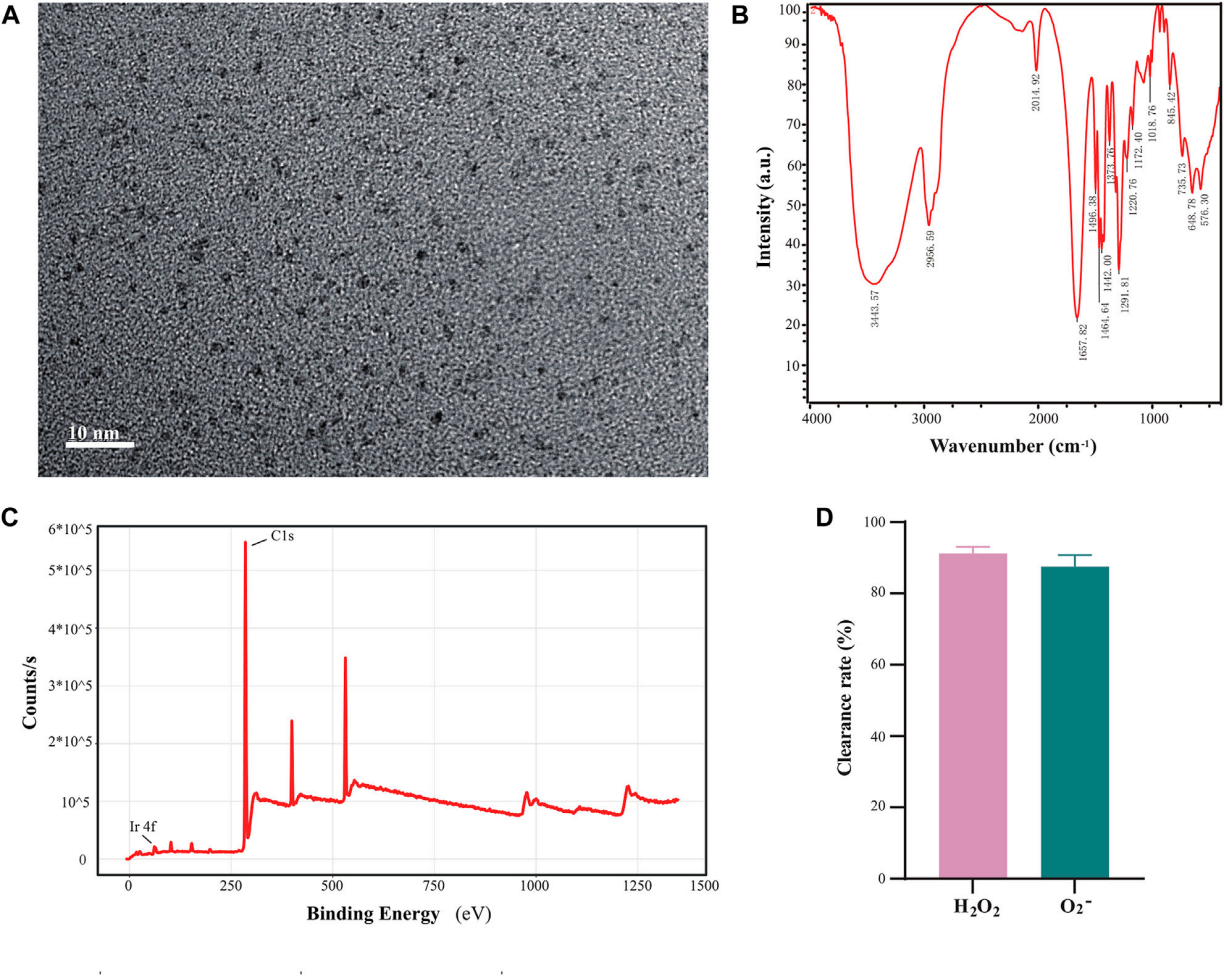
Synthesis of polyvinyl pyrrolidone (PVP) modified iridium nanoparticles. **(A)** Transmission electron microscopy characterization of iridium nanoparticles (scale bar, 10 nm). **(B)** Infrared spectroscopic characterization of ultra-small iridium nanoparticles. **(C)** X-ray photoelectron spectroscopy (XPS) characterization of ultra-small iridium nanoparticles. **(D)** Detection of the enzymatic activity of 1 mg/mL ultra-small iridium nanoparticles.

We synthesized and characterized ultra-small Ir-NPs with enzyme-like activity using a previously mentioned method with modifications. PVP was used to modify iridium chloride, and ultra-small iridium nanoparticles with particle sizes between 1 and 2 nm were synthesized according to the TEM characterization results (Figure 1A). The XPS results showed the presence of Ir in the synthesized Ir-NPs (Figure 1C). FT-IR spectra revealed the presence of peaks corresponding to the C-N and C=O bonds at 1,291 and 1,657 cm^−1^, respectively, and the presence of PVP on the surface of the Ir-NPs was identified (Figure 1B). These results were consistent with the literature (Zhang et al., 2021a). In addition, the simulated SOD and CAT enzymatic activities were evaluated based on the removal rates of superoxide anion and hydrogen peroxide. It was found that 1 mg/mL of ultra-small iridium nanoparticles could successfully remove 87.5% ± 3.2% of •O^2−^ and 91.2% ± 1.9% of H_2_O_2_, which indicated that Ir-NPs exhibits SOD and CAT mimic catalytic activities (Figure 1D).

### 3.2 Ir-NPs can improve kidney injury caused by acute exposure to high altitude-induced hypoxia

**FIGURE 2:**
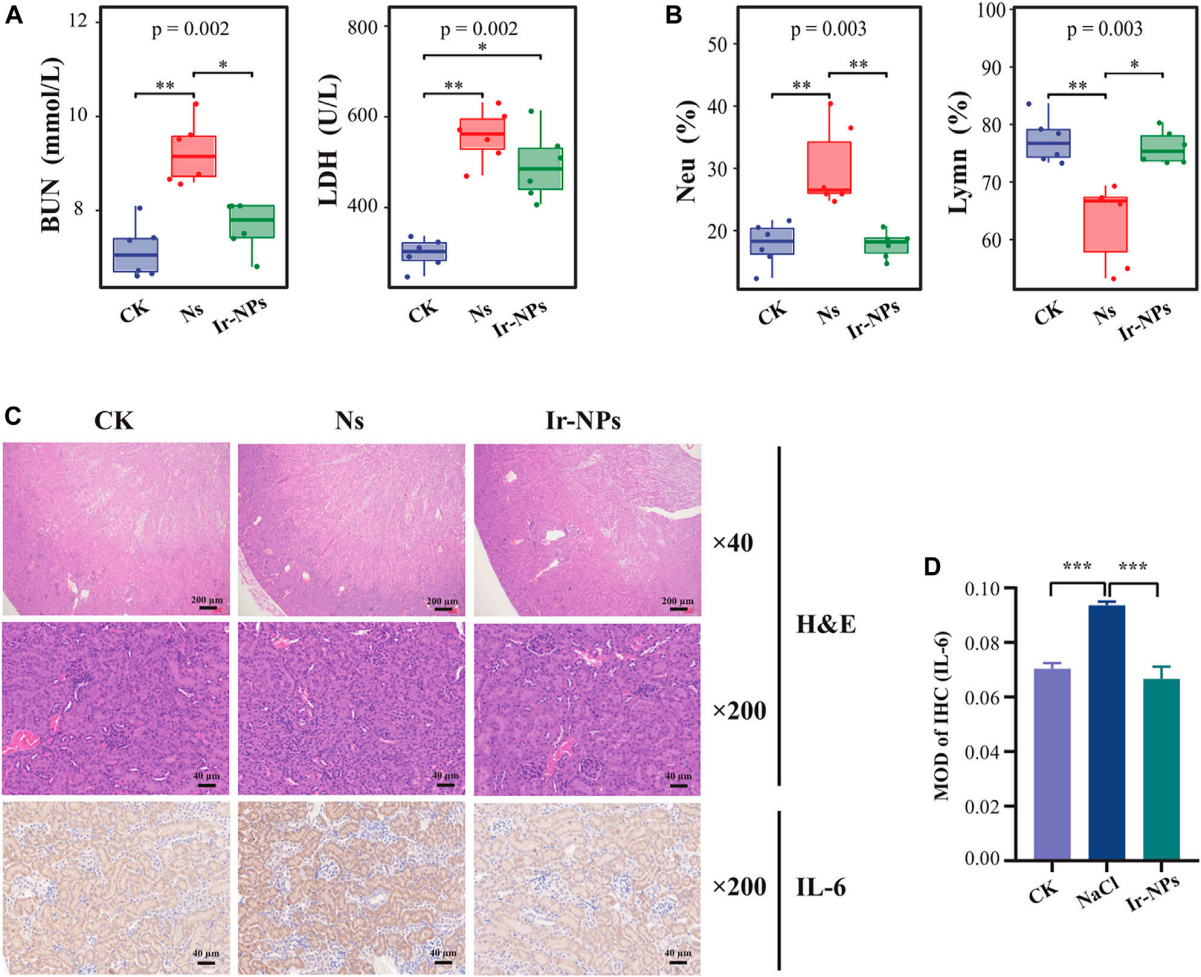
Effect of Iridium nanozymes (Ir-NPs) on kidney injury. **(A)** Plasma biochemical test. The Kruskal—Wallis test was used to obtain the overall *p*-value, and the difference between the three groups was statistically significant (*p* < 0.05). Dunn’s pairwise comparison test was used to obtain the statistical significance of the differences in plasma lactate dehydrogenase (LDH) and urea nitrogen (BUN) (*, *p* < 0.05; **, *p* < 0.01). **(B)** Blood cell count. Kruskal—Wallis test was used to obtain the overall *p*-value, and the difference between the three groups was statistically significant (*p* < 0.05). Dunn’s pairwise comparison test was used to obtain the statistical significance of the differences in the proportion of lymphocytes (Lym %) and the proportion of neutrophils (Neu%) (*, *p* < 0.05; **, *p* < 0.01). **(C)** H&E staining of representative sections of mouse kidneys and immunohistochemistry of IL-6 expression in the mice kidneys in each treatment group. **(D)** Analysis of the average optical density related to IL-6 expression in each group. One-way analysis of variance (ANOVA) was used to compare the average optical density among groups, and the difference between each two groups was statistically significant (*p* < 0.001).

The changes in the physiological and biochemical indices of mice in the different treatment groups were analyzed after intragastric administration of Ir-NPs. Among the mouse biochemical indices analyzed, blood urea nitrogen (BUN) and lactate dehydrogenase (LDH) levels were significantly increased in the Ns group (*p* < 0.01) compared with that of the CK group; however, BUN and LDH levels were significantly decreased in the Ir-NPs group (*p* < 0.05) (Figure 2A). This suggests that Ir-NPs can alleviate inflammation and renal function decline caused by acute exposure to high altitude-induced hypoxia. In addition, the proportion of lymphocytes (Lym %) was significantly lower in the Ns group (*p* < 0.01), while the proportion of neutrophils (Neu%) was significantly higher (*p* < 0.01) when compared with the CK group. In contrast, the proportion of these cells was restored in the Ir-NPs group, suggesting that Ir-NPs could affect body immunity and reduce inflammation (Figure 2B). In addition, there was a significant increase in total protein, aspartate aminotransferase, and creatine kinase levels in the Ns group (*p* < 0.05); however, there was a decreasing trend in the Ir-NPs group (Supplementary Figure S1A). In the blood cell tests, the Ns group had a significant decrease in WBCs (white blood cells) and Lym % (*p* < 0.01) (Supplementary Figure S1B).

Compared to the normal CK group, histopathological staining of mouse kidneys showed glomerular atrophy, vascular endothelial hyperplasia, local edema of the renal tubule cell space, interstitial vascular dilation and congestion, and infiltration of a few inflammatory cells in the Ns group. The kidney tissue structure was reversed in the Ir-NP group, as was the cortical medulla boundary; the glomerulus and renal tubule cell staining was uniform, the interstitial and congested blood vessels were dilated, and the number infiltrating inflammatory cells were reduced. In addition, the immunohistochemical staining results showed that the expression of IL-6 in the kidney tissue of mice in the Ns group was higher than in the CK group. In contrast, the expression of IL-6 was considerably downregulated in the Ir-NPs group compared with the Ns group (Figures 2C, D). The results of these experiments show that Ir-NPs can ameliorate kidney injury to a certain extent under acute exposure to simulate high-altitude hypoxia conditions.

### 3.3 Ir-NPs can regulate intestinal flora

We used 16S rRNA sequencing to detect the changes in microbial composition in the intestinal samples. After the uchime method was used for QC, denoising, splicing, removing chimerism, and singleton OTUs, 389,783 high-quality data (valid labels) were generated from nine samples (mean: 43,309), and 983 OTUs were obtained at a 97% similarity level. Most of the common genera belong to *p-Firmicutes*. There were 96, 123, and 47 unique OTUs in the CK, Ns, and Ir-NP groups, respectively, and 375 OTUs were shared between them.

**FIGURE 3:**
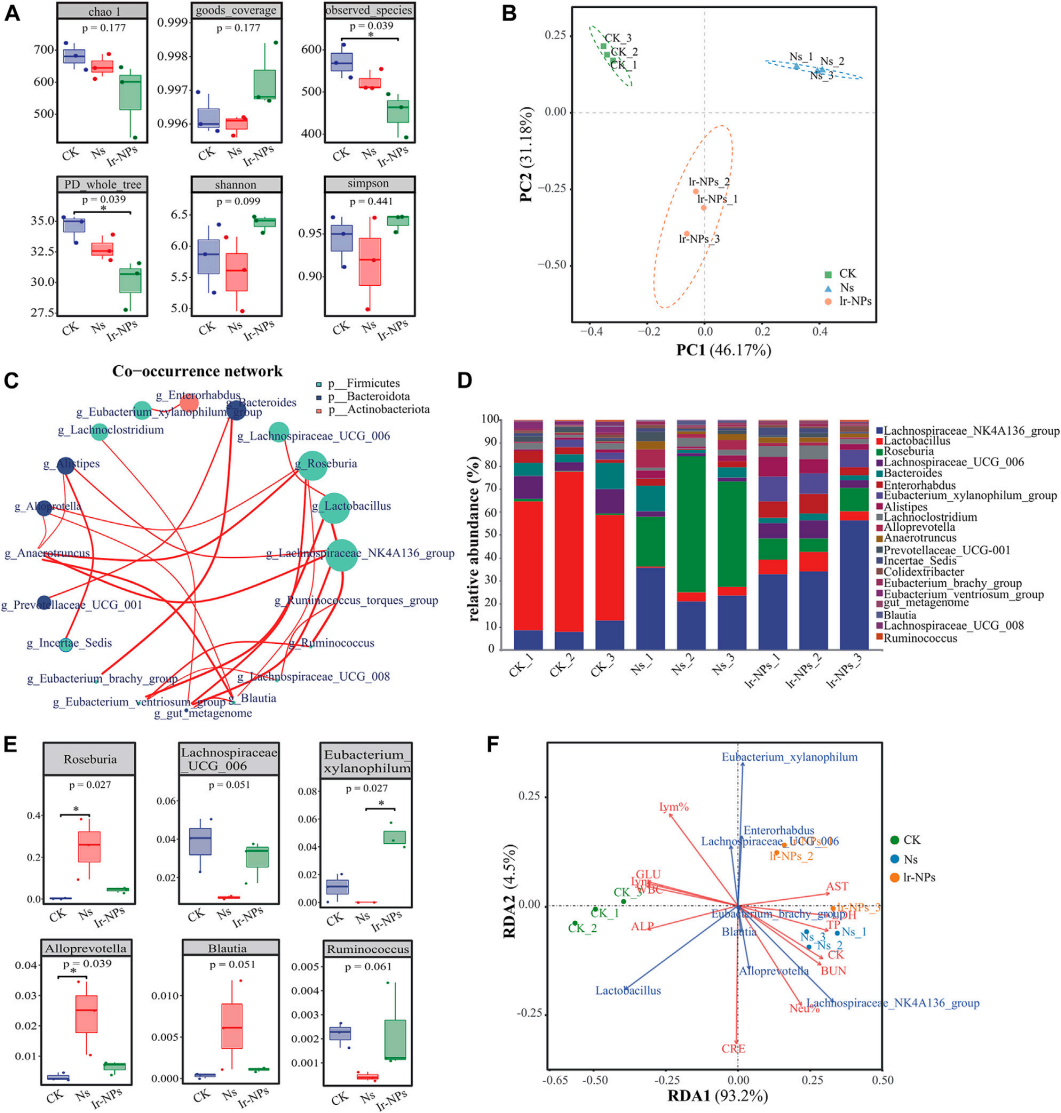
Distribution and intergroup differences in the microbial communities between treatment groups. **(A)** α diversity. The coverage index was calculated for 983 OTUs across nine samples. **(B)** Weighted UNIFRACPCOA chart. **(C)** Relative abundance analysis at the phylum level. The size of the dot represents the abundance, the thickness of the line represents the correlation, and the color of the dots represents the door they belong to. **(D)** Superimposed map of species composition at the genus level. Different colors indicate different families and genera. **(E)** Changes in the relative abundance of six species of bacteria at the genus level. The degree of influence of these species was significantly different among different groups (*p* < 0.05). **(F)** RAD correlation analysis. Dots represent different samples, arrows represent different biochemical and physiological indicators, and angles represent correlations.

The α diversity of the mouse microbial communities in the various treatment groups was analyzed using the Chao1, Simpson, Shannon, PD_whole_tree, Observed_species, and Goods_coverage indices. These indices represent the diversity, richness, and evenness of the microbial flora in the mice. There were no significant differences in six α-diversity indices among the different groups (*p* > 0.01), indicating that the species richness and evenness of the different groups were similar and tended to be stable (Figure 3A). Further β diversity analysis showed that the bacterial community structure of the CK, Ns, and Ir-NPs groups had significant differences in phylogenetic distance. PCoA (Principal co-ordinates analysis) showed that different treatments significantly changed the microbial diversity in the intestinal contents of the mice (Figure 3B).

Microbial co-occurrence networks are widely used to explore connections in microbial communities. Microbial species abundance was calculated using the Spearman inspection method, and all samples were selected for genus-level correlation analysis. The corresponding doors were used as a legend, the calculation results were filtered to values of |R|<0.2 for drawing. Analysis of the relative microbial abundance at the genus level showed that *g-Lachnospiraceae-NK4A136_group*, *g-Lactobacillus*, *g-Roseburia*, *g-Lachnospiraceae_UCG_006* under *p-Firmicutes*, and *g-Bacteroides* under *p-Bacteroidetes* gate had a higher abundance in general (Figure 3C).

Further, when the changes in microbial composition were analyzed at the genus level, *Lachnospiraceae_NK4A136_group* and *Bacteroides* showed an increase in relative abundance, while *Lactobacillus* decreased in the Ns and Ir-NP groups (Figure 3D). The *Lachnospiraceae_NK4A136_group, Enterorhabdus, Eubacterium_xylanophilum_group*, and *Lachnospiraceae_UCG_006* in the Ir-NPs group may become the dominant bacterial groups by competing against other bacteria. In the Ns group, the analysis between the horizontal groups revealed that *Roseburia, Alloprevotella,* and *Blautia* showed an obvious increase in abundance, while *Lachnospiraceae_UCG_006, Eubacterium_xylanophilum,* and *Ruminococcus* showed an apparent decrease in abundance. These changes in the Ns group were mitigated by the Ir-NPs treatment (Figure 3E). According to the RAD analysis results, *Roseburia, Alloprevotella*, and *Blautia* were the characteristic bacteria under exposure to acute altitude hypoxia, and they had a latent correlation with this model. Meanwhile, *Enterorhabdus*, *Eubacterium_xylanophilum_group*, and *Lachnospiraceae_UCG_006* were the characteristic bacteria in the Ir-NPs group (Figure 3F).

### 3.4 Correlation of the microbial genera to the physiological and biochemical parameters and analysis of their functions

**FIGURE 4:**
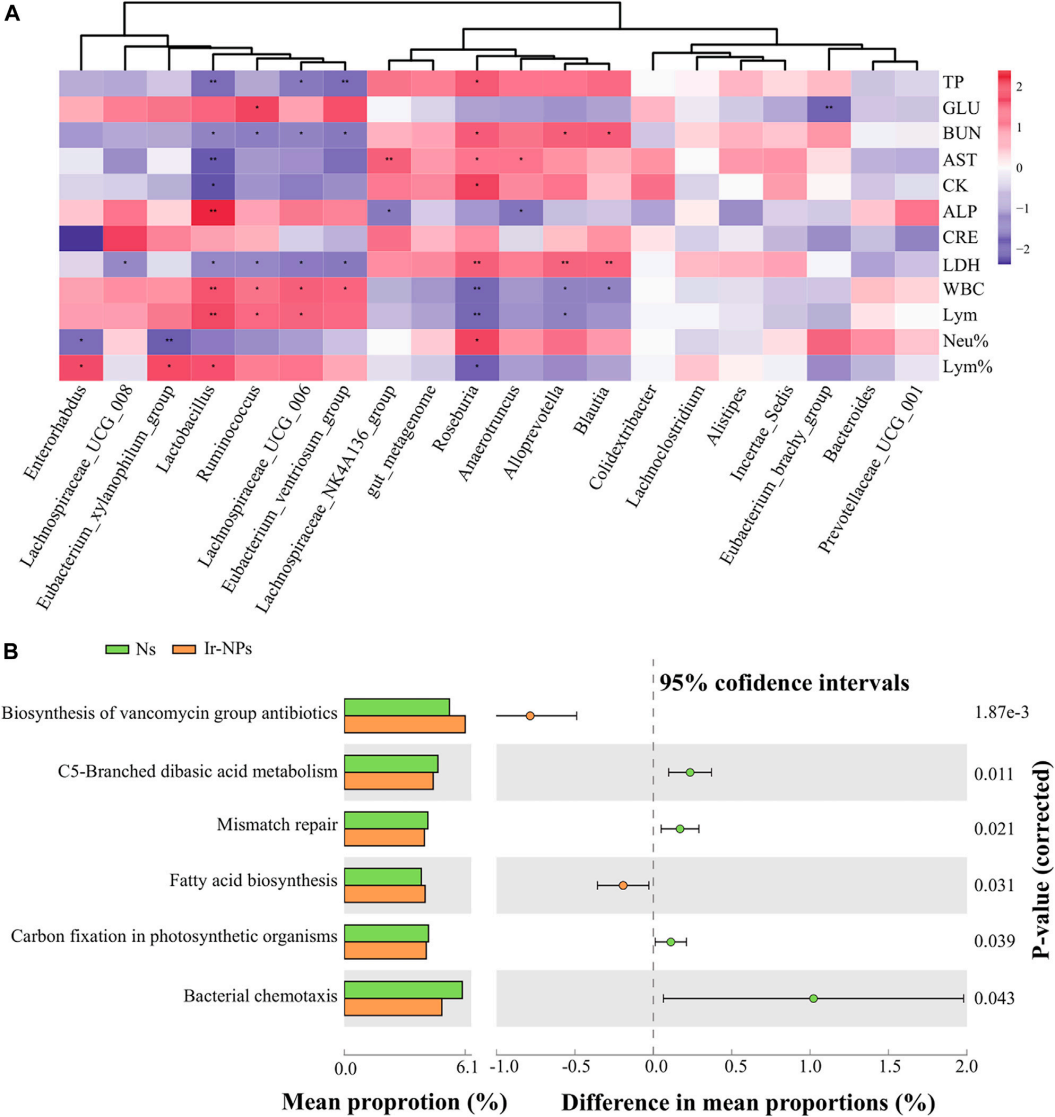
Correlation between physiological and biochemical parameters and functional analysis of microbial genera. **(A)** Heat map analysis of the correlation between the top 20 most abundant genera and physiological and biochemical indices (*, *p* < 0.05; **, *p* < 0.01) **(B)** Functional prediction analysis of the most abundant genera using picrust2.

Heat maps were generated to analyze the correlation between the changes in the biochemical indices and the main gut flora. According to the correlation analysis between biochemical indicators and bacterial abundance at the genus level, LDH and BUN were positively correlated with *Roseburia, Alloprevotella,* and *Blautia,* and negatively correlated with *Lactobacillus, Lachnospiraceae_UCG_006, Eubacterium_ventriosum_group,* and *Ruminococcus*. Lym% was negatively correlated with *Roseburia*, while it was positively correlated with *Lactobacillus*, *Eubacterium_xylanophilum-group,* and *Enterorhabdus*. Neu% was positively correlated with *Roseburia* and negatively correlated with *Eubacterium_xylanophilum* and *Enterorhabdus* (Figure 4A). In addition, the flora metabolic pathways were preliminarily predicted, and the top 20 bacteria genera in the Ns group were compared with that of the Ir-NPs group. The results showed differences in the following six pathways: biosynthesis of vancomycin, C5-branched dibasic acid metabolism, mismatch repair, fatty acid biosynthesis, carbon fixation in photosynthetic organisms, and bacterial chemotaxis (Figure 4B).

### 3.5 Analysis of plasma metabolic maps and metabolite characteristics in the different groups of mice

**FIGURE 5:**
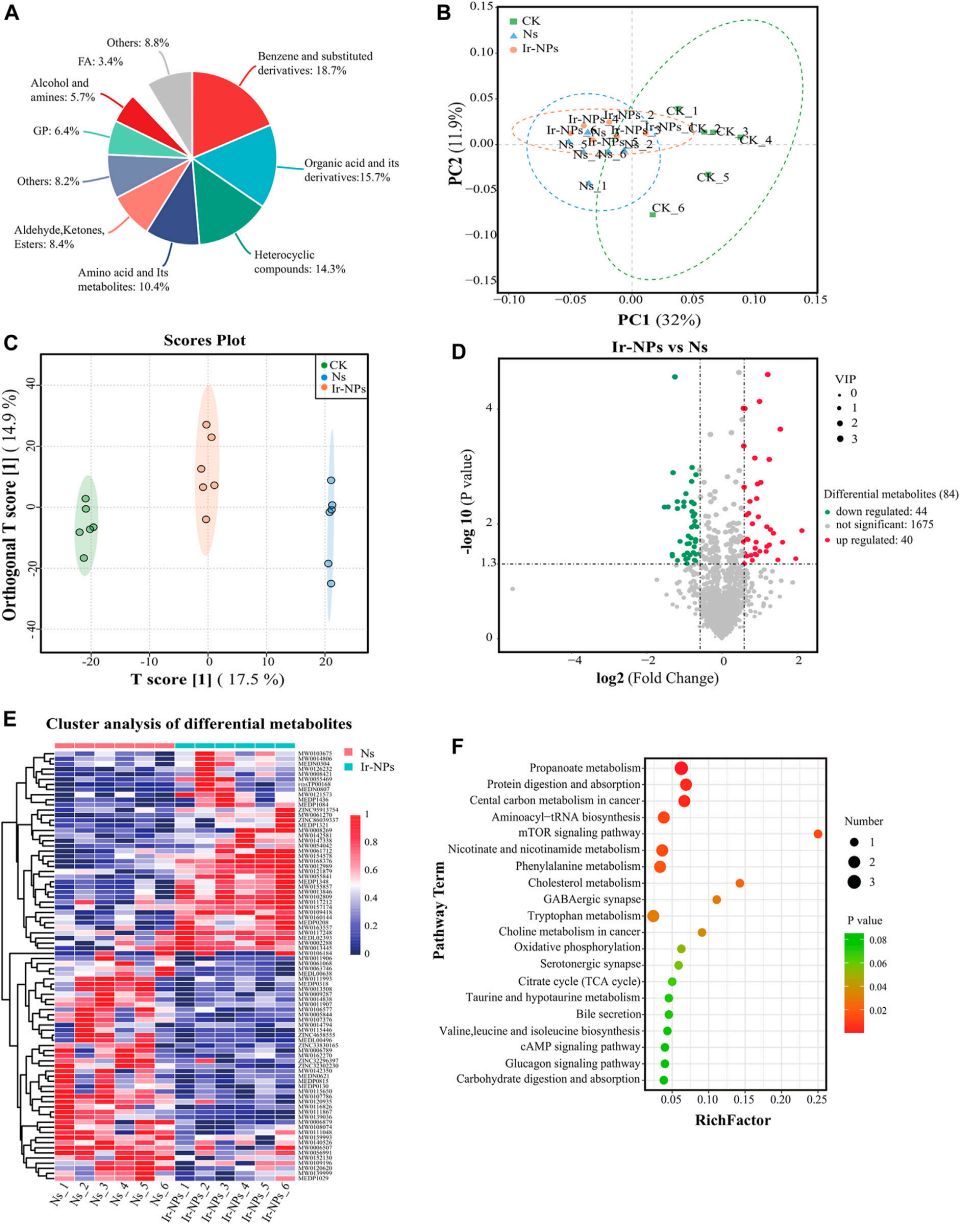
Analysis of plasma metabolic profiles and metabolite characteristics. **(A)** Analysis of the plasma metabolite composition in mice. **(B)** Weighted UNIFRACPCOA. **(C)** Orthogonal partial least squares discriminant analysis (OPLS-DA). **(D)** Volcano plots of the differential metabolites between the Ir-NPs and Ns groups. **(E)** Cluster analysis of 84 different metabolites. **(F)** KEGG analysis. The size of the dot represents the enrichment of the differential metabolites.

In this study, non-targeted metabolomics analysis using UPLC-MS/MS was used to study the plasma metabolites in the different treatment groups. Based on the secondary mass measurement results, a total of 1,858 metabolites were annotated. The top five categories were benzene and its substituted derivatives; organic acids and their derivatives; heterocyclic compounds; amino acids and their metabolites; and aldehydes, ketones, and esters, among others (Figure 5A). The PCoA shows significant differences between the Ns and CK groups (Figure 5B). According to the commonly used model of metabolite difference, OPLS-DA, this model has good stability and high reliability. Hence, the model was used to analyze differences in plasma metabolites in the Ir-NPs, Ns, and CK groups. Each treatment group was distributed independently without overlap and was significantly different from the control group (Figure 5C). From the perspective of the volcano plot of the differential metabolites between the Ir-NPs and Ns groups and according to the threshold parameters *p* < 0.05, |log FC| > 1, and VIP >1, a total of 84 metabolites were obtained. Compared with the Ns group, 44 metabolites were upregulated and 40 were downregulated in the Ir-NPs group (Figure 5D).

Cluster analysis of the Ns and Ir-NPs groups showed significant differences in metabolite abundance (Figure 5E). To investigate the metabolic response mechanism of Ir-NPs-administered mice, KEGG enrichment analysis of metabolites to identify potential metabolic pathways that were enriched after treatment was performed. Differential metabolites between Ns and Ir-NPs group were significantly enriched in propanoate metabolism, protein digestion and absorption, central carbon metabolism in cancer, aminoacyl-tRNA biosynthesis, mTOR signaling pathway, nicotinate and nicotinamide metabolism, and phenylalanine metabolism. Among these, the number of differential metabolites associated with propionic acid metabolism was the highest (Figure 5F).

### 3.6 Correlation analysis between the microbiome, metabolites, and physiologic and biochemical parameters

**FIGURE 6:**
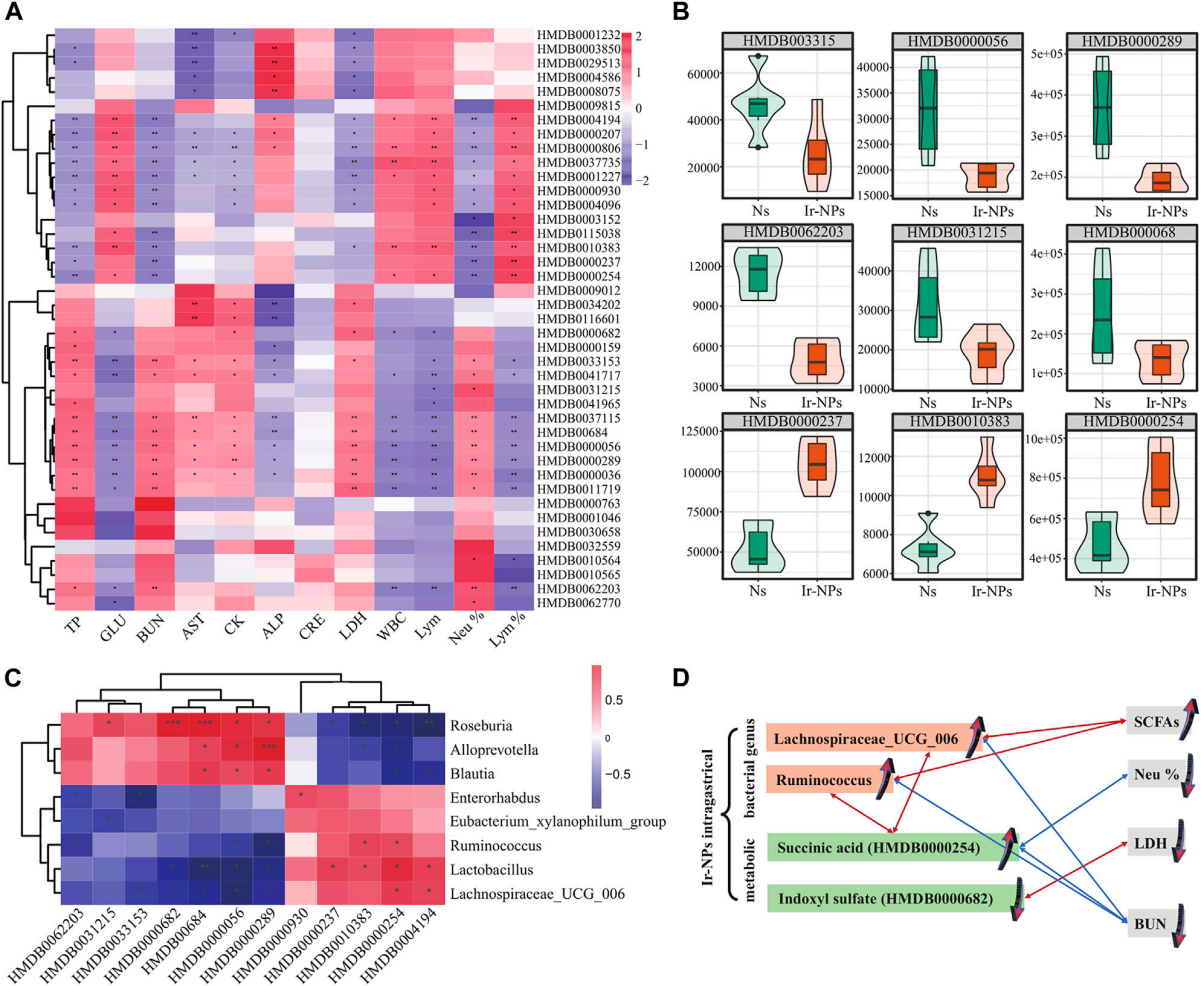
Correlation analysis between differential metabolites and physiological and biochemical indicators. **(A)** Correlation analysis between differential metabolites and 12 physiological and biochemical indicators. Asterisks indicate statistical significance (*, *p* < 0.05; **, *p* < 0.01). **(B)** Violin diagram of the differential metabolites. The ordinate indicates the relative content of differential metabolites. **(C)** Combined analysis of microbial metabolites (*, *p* < 0.05). **(D)** Schematic diagram of the relationship between metabolic, bacterial genus and blood indicators. A red line means they are positively correlated, and a blue line means they are negatively correlated. Arrows direction up or down represent the increase and decrease of expression level, respectively.

Finally, we analyzed the correlation between physiological and biochemical indices, microbiota, and major differential metabolites. Results of the cluster analysis among physiological and biochemical indicators and metabolites showed that succinic acid had a significant negative correlation with BUN and Neu% (*p* < 0.01); and there was a significant positive correlation between indoxyl sulfate and LDH (*p* < 0.05) (Figure 6A). Among the differential metabolites of the Ns and the Ir-NPs groups, (Z)-4′,6-Dihydroxyaurone, beta-alanine, uric acid, L-kynurenine, triiodothyronine glucuronide, N-ethylacetamide, and indoxyl sulfate were upregulated in the Ir-NPs group; in contrast, succinic acid, propionic acid, Lyso-PC, N1-methyl-4-pyridone-3-carboxamide, and trans-cinnamic acid were downregulated (Figure 6B). The details of the metabolites are listed in Table 1. A joint analysis of the microbiota and metabolites showed that succinic acid was significantly positively correlated with *Ruminococcus* and *Lachnospiraceae_UCG_006* (Figure 6C). When synthesizing all joint analyses (Figure 6D), the abundance of *Lachnospiraceae_UCG_006* and *Ruminococcus* was increased in the Ir-NPs group. These two genera were positively correlated with short-chain fatty acids (SCFAs) and negatively correlated with BUN levels. In addition, the increase in succinic acid levels in the Ir-NPs group was negatively correlated with Neu% and BUN, whereas the decrease in indoxyl sulfate levels was positively correlated with LDH levels. These combined effects show the possible reasons for the reduction in inflammation and amelioration of kidney injury.

**TABLE 1:** Information on the differential metabolites. Compared with the mice subjected to an environment with typical oxygen levels, there were nine significantly different metabolites in mice subjected to high altitude-induced hypoxia.

HMDB ID	Common name	Chemical formula	Super class	Class	Sub class
HMDB0033153	(Z)-4′,6-Dihydroxyaurone	C_15_H_10_O_4_	Phenylpropanoids and polyketides	Aurone flavonoids	NA
HMDB0000056	beta-Alanine	C_3_H_7_NO_2_	Organic acids and derivatives	Carboxylic acids and derivatives	Amino acids, peptides, and analogs
HMDB0000289	Uric acid	C_5_H_4_N_4_O_3_	Organoheterocyclic compounds	Imidazopyrimidines	Purines and purine derivatives
HMDB0062203	Triiodothyronine glucuronide	C_21_H_20_I_3_NO_10_	Organic acids and derivatives	Carboxylic acids and derivatives	Amino acids, peptides, and analogs
HMDB0031215	N-Ethylacetamide	C_4_H_9_NO	Organic acids and derivatives	Carboximidic acids and derivatives	Carboximidic acids
HMDB0000682	Indoxyl sulfate	C_8_H_7_NO_4_S	Organic acids and derivatives	Organic sulfuric acids and derivatives	Arylsulfates
HMDB0000254	Succinic acid	C_4_H_6_O_4_	Organic acids and derivatives	Carboxylic acids and derivatives	Dicarboxylic acids and derivatives
HMDB0000237	Propionic acid	C_3_H_6_O_2_	Organic acids and derivatives	Carboxylic acids and derivatives	Carboxylic acids
HMDB0010383	LysoPC	C_24_H_48_NO_7_P	Lipids and lipid-like molecules	Glycerophospholipids	Glycerophosphocholines

## 4 Discussion

The distribution of microorganism changes in a plateau environment, and this imbalance promotes the bacterial production of uremic toxins. These metabolites, most of which are excreted by the kidney, enter the bloodstream through the damaged intestinal barrier, and their retention within the kidney leads to renal dysfunction (Nallu et al., 2017). The plasma biochemical indices of the mice revealed that urea nitrogen levels were significantly increased in the model group. Over 90% of urea nitrogen, a protein catabolic byproduct, is excreted through the kidneys in the human body, with the remainder excreted through the gut and skin. When the BUN levels increase, various kidney lesions occur, impairing its normal excretory function. Blood BUN concentration is considered a traditional marker of kidney function (Li et al., 2019). These findings suggest that mice may have renal injury upon exposure to a high altitude. However, the urea nitrogen level of mice in the Ir-NP group was restored, suggesting that Ir-NPs can alleviate the decline in renal function caused by acute altitude hypoxia.

Kidney damage is often induced by several factors. In recent years, there has been much interest in the relationship between the gut microbiota and kidney diseases. The immunomodulatory role of intestinal flora in kidney diseases has also been reported. The gut microbiota plays a significant role in host health, and without gut microbiota, normal immune development and function are impaired. Numerous studies have shown that acute exposure to simulated high-altitude hypoxia alters the gut microbiota of mice (Wang et al., 2022a; Lin et al., 2022). Exposure to hypoxia can increase the risk of abdominal pain, infection, acute mountain sickness, and even gastrointestinal illness. Studies in rodents have shown that dynamic changes in the gut microbiota may contribute to the above conditions. Exposure to hypoxia increases the risk of GI (gastrointestinal tract) damage, oxidative stress, and GI permeability, which are accompanied by changes in the gut microbiota composition and activity (Adak et al., 2014). In this study, the analysis of genus-level differences between groups showed that Ir-NPs decreased the increased abundance of *Roseburia, Alloprevotella,* and *Blautia*, as well as increased the decreased abundance of *Lachnospiraceae_UCG_006, Eubacterium_xylanophilum*, and *Ruminococcus* upon exposure to acute altitude hypoxia. The characteristic bacterium *Ruminococcus* in the Ir-NPs group was positively correlated with SCFAs and has been reported to improve inflammation and intestinal diseases (Wen et al., 2004). SCFAs can exert systemic effects after entering the bloodstream, such as increasing the secretion of anti-inflammatory factors and downregulating autoimmune-related factors (Rooks and Garrett, 2016). Compared with the CK group, *Lachnospiraceae_UCG_006* had a lower abundance in the Ns group and was positively associated with SCFAs. There are currently few reports on *Eubacterium_xylanophilum*. In a mouse antibiotic-related diarrhea model, the abundance of *Eubacterium_xylanophilum* decreased; however, it improved after probiotics treatment (Huang et al., 2022). Intragastric administration of Ir-NPs in mice could restore the decline in the abundance of beneficial bacteria caused by acute altitude hypoxia. Lactate dehydrogenase (LDH) is a plasma enzyme widely present in various human tissues. When inflammation and necrosis occur in the organs or tissues, this enzyme is released into the blood, increasing plasma LDH levels. In the present study, the elevation of LDH levels in the model group suggests that a rapid elevation to higher altitudes could cause inflammation; however, Ir-NPs could reduce this tendency.

Inflammatory responses are frequently reported in studies of acute altitude hypoxia. Plasma levels of IL-1β, IL-6, and TNF-α were found to be increased in individuals with altitude sickness compared to controls in low-altitude environments (Liu et al., 2017; Wang et al., 2018). Other studies have shown that certain inflammatory mediators are upregulated upon exposure to high altitudes or under acute hypoxia, even in the absence of infection (Pham et al., 2021). Conversely, some studies have reported that the downregulation of proinflammatory factors can help individuals in plain areas adapt to high altitudes (Yi et al., 2021). In this study, Ir-NPs reduced the increase in IL-6 expression level in mice exposed to acute altitude hypoxia. These results suggest that Ir-NPs may help mice adapt to rapid entry in a high-altitude environment. Simultaneously, the proportion of neutrophils also increased in the model group, reflecting the inflammatory response under acute altitude hypoxia; however, the administration of Ir-NP recovered the increased proportion of neutrophils. These results suggest that Ir-NPs can reduce the inflammatory response, affect body immunity, and ameliorate kidney injury. In the correlation analysis between biochemical indices and bacteria at the genus level, BUN was negatively correlated with *Lactobacillus, Lachnospiraceae_UCG_006, Eubacterium_ventriosum_group*, and *Ruminococcus*. When the results of the biochemical analyses were combined, BUN levels increased in the Ns group while they decreased in the Ir-NPs group, consistent with the findings that BUN was negatively correlated with *Lachnospiraceae_UCG_006* and *Ruminococcus*. These results suggest that Ir-NPs can decrease BUN levels by regulating the increase in the abundance of *Lachnospiraceae_UCG_006* and *Ruminococcus*, reduce inflammation, and improve renal function by producing SCFAs. Hypoxia exposure can alter microbiome composition, which may significantly impact the metabolites produced, and there is a correlation between the two. In this study, functional prediction of the intestinal microbiota revealed that between the Ns and Ir-NPs groups, the main pathway of C5-branched dicarboxylic acid metabolism was related to fatty acid biosynthesis. In other words, the differences between the Ns and Ir-NPs groups are primarily related to the synthesis and metabolism of fatty acids, suggesting that Ir-NPs affect the production of bacterial metabolites by affecting bacterial distribution, resulting in functional differentiation of the metabolites produced, causing further inflammation and biochemical changes, and improving renal injury.

Several studies have shown that the gut microflora produces many metabolites in the host blood. Recent metabolomic studies have revealed a strong link between gut microbial sources, endogenous host metabolites, and various diseases (Guasch-Ferre et al., 2016). The gut-kidney axis theory can explain the effector relationship between the gut microbiota and metabolites in renal injury. Therefore, impaired kidney function may lead to disturbances in the gut microbiota (Sabatino et al., 2015). Long-term hypoxia may damage the intestinal epithelial tissue, destroy the tight junctions between these cells, and produce harmful substances such as endotoxins. Damage to the intestinal epithelium can translocate endotoxins and bacterial metabolites from the intestine, enter the blood circulation through the damaged intestinal barrier, cause inflammation, and accelerate kidney injury (Meijers et al., 2018; Borges and Mafra, 2019).

Of the 80 differential metabolites we found that were associated with biochemical indicators, approximately 40 were found in the Human Metabolic Database. Among them, (Z)-4′,6-Dihydroxyaurone (HMDB0033153), beta-alanine (HMDB0000056), N-ethylacetamide (HMDB0031215), and indoxyl sulfate (HMDB0000682) were downregulated in the Ir-NPs-treated group compared to the Ns group. In contrast, succinic acid (HMDB0000254), propionic acid (HMDB0000237), and LysoPC (HMDB0010383) were upregulated in the Ir-NPs-treated group compared with the Ns group. (Z)-4′,6-Dihydroxyaurone and N-ethylacetamide mainly play a role as defense or signaling molecules, whereas HMDB0000056, HMDB0000254, and HMDB0000237 primarily focus on propionate metabolism. Related flora can regulate metabolite production and inflammatory reactions to affect renal function, according to the results of our metabolite analysis. Succinic acid levels were significantly higher in the Ir-NP group than in the Ns group. Succinate has a variety of biological roles, including as a metabolic intermediate and a cell signaling molecule. Succinate links cellular metabolism, particularly ATP formation, to the regulation of cellular function. Succinate can be broken down or metabolized by succinate dehydrogenase to produce fumarate, a part of the electron transport chain that generates ATP. In humans, succinic acid is produced by *Enterobacter*, which is classified as *Enterorhabdus.* However, *Enterorhabdus* was negatively correlated with the Neu% in this study. It is suggested that the increase in succinic acid metabolism in the Ir-NPs group decreased Neu%, improved the inflammatory response, and reduced kidney injury. Indoxyl sulfate (HMDB0000682) is also a bacterial co-metabolite. In humans and other mammals, high levels of indoxyl sulfate in the whole blood or plasma are associated with developing and progressing chronic kidney disease and cardiovascular disease. Indoxyl sulfate is a renal tubular toxin that can induce apoptosis and necrotic cell death in renal tubular cells. It upregulates signal transducers and activators of transcription-3 phosphorylation, leading to increased production of TGF-β1, monocyte chemo protein-1, and α-smooth muscle actin, all of which are involved in interstitial inflammation, renal fibrosis, and kidney disease progression. Hence, as a uremic toxin, indoxyl sulfate stimulates glomerulosclerosis and interstitial fibrosis, hastening the progression of renal failure. These findings are consistent with those of the current study. Moreover, indoxyl sulfate was positively correlated with LDH levels in our correlation analysis. Therefore, the reduction in indoxyl sulfate levels in the Ir-NPs group also reduced LDH levels, improved the inflammatory response, and reduced renal injury.

Based on the above correlation analysis, Ir-NPs changed the distribution of microbial communities and the metabolism of propionic acid under rapid altitude elevation. This further affected the changes in the levels of metabolites in the blood, and the physiological and biochemical indices through the entero-renal axis. After administration of Ir-NPs, the characteristic bacteria *Lachnospiraceae_UCG_006* and *Ruminococcus* increased in abundance, which in turn increased the production of SCFAs; however, these bacterial groups were negatively correlated with BUN levels. Meanwhile, *Ruminococcus* was positively correlated with succinic acid, whereas succinic acid was negatively correlated with Neu%. Indoxyl sulfate was positively correlated with LDH. At the same time, a decreased Neu%, LDH and BUN levels, and IL-6 expression in mice was detected. From these results, we speculated that inflammation was reduced and kidney damage was relieved in mice treated with Ir-NPs.

In our study, we discovered that Ir-NPs can reduce the inflammatory response and protect kidney function under acute altitude hypoxia, which may be related to intestinal flora distribution regulation and plasma metabolism in mice. Our results provide a new treatment strategy for kidney injury caused by hypoxia at high altitude.

## Data Availability

The raw data supporting the conclusion of this article will be made available by the authors, without undue reservation.
