# Supplementary material for: Protective effect of bioactive iridium nanozymes on high altitude-related hypoxia-induced kidney injury in mice
Source: Front Pharmacol. 2023 Feb 20;14:1115224. doi: 10.3389/fphar.2023.1115224 (PMC9986433; doi:10.3389/fphar.2023.1115224)
Supplement: Supplementary file 1 [file DataSheet1.DOCX]

Supplementary Material

# Supplementary Figure


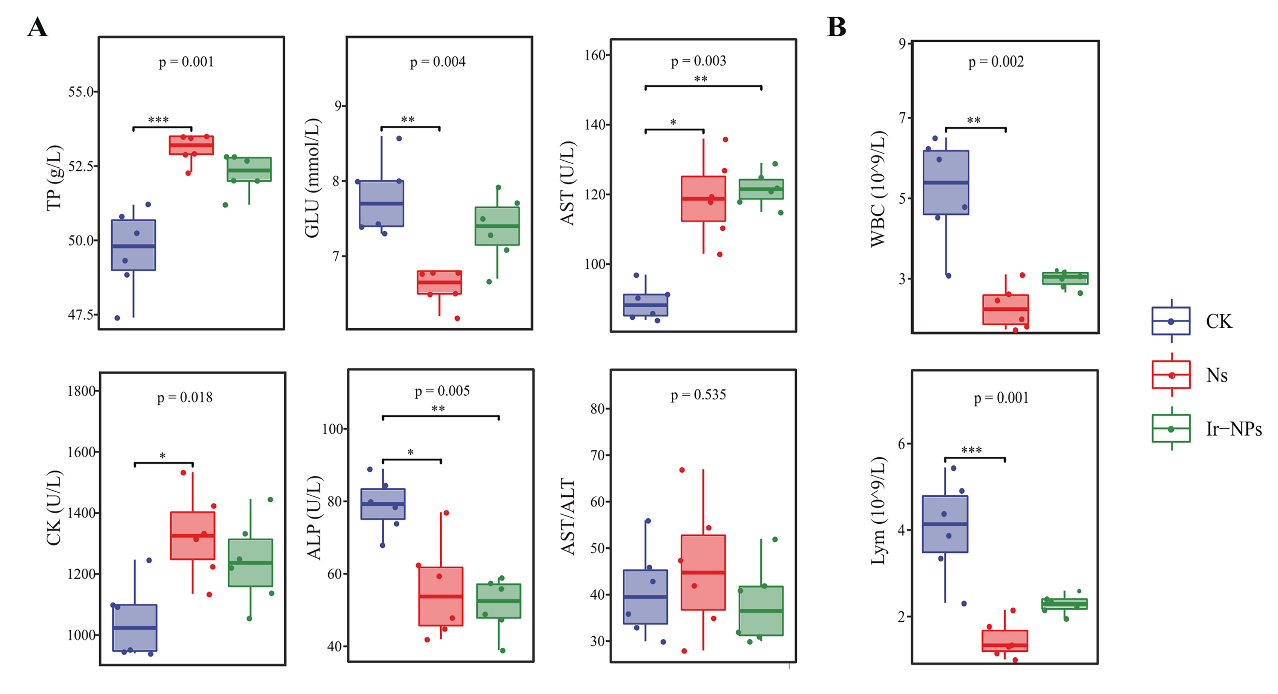


**Supplementary Figure 1.** Physiological and biochemical indicators: (A) Biochemical changes. (B) Physiological changes. The Kruskal–Wallis test was used to obtain the overall P-value, and the difference between the three groups was statistically significant (*p*<0.05).
